# Overview of North American Isolates of Chronic Wasting Disease Used for Strain Research

**DOI:** 10.3390/pathogens14030250

**Published:** 2025-03-04

**Authors:** W. David Walter, Allen Herbst, Chia-Hua Lue, Jason C. Bartz, M. Camille Hopkins

**Affiliations:** 1 U.S. Geological Survey, Pennsylvania Cooperative Fish and Wildlife Research Unit, The Pennsylvania State University, University Park, PA 16802, USA; 2 U.S. Geological Survey, National Wildlife Health Center, Madison, WI 53711, USA; aherbst@usgs.gov; 3 Pennsylvania Cooperative Fish and Wildlife Research Unit, The Pennsylvania State University, University Park, PA 16802, USA; chiachia926@gmail.com; 4 Department of Medical Microbiology and Immunology, Creighton University, Omaha, NE 68178, USA; jbartz@creighton.edu; 5 U.S. Geological Survey, Ecosystems Mission Area, Reston, VA 20192, USA; mchopkins@usgs.gov

**Keywords:** chronic wasting disease, prion strains, tissue, infected isolates, transmission

## Abstract

Chronic Wasting Disease (CWD) is a prion disease that affects *Cervidae* species, and is the only known prion disease transmitted among wildlife species. The key pathological feature is the conversion of the normal prion protein (PrP^C^) misfolding into abnormal forms (PrP^Sc^), triggering the onset of CWD infections. The misfolding can generate distinct PrP^Sc^ conformations (strains) giving rise to diverse disease phenotypes encompassing pathology, incubation period, and clinical signs. These phenotypes operationally define distinct prion strains, a pivotal element in monitoring CWD spread and zoonotic potential—a complex endeavor compounded by defining and tracking CWD strains. This review pursues a tripartite objective: 1. to address the intricate challenges inherent in ongoing CWD strain classification; 2. to provide an overview of the known CWD-infected isolates, the strains they represent and their passage history; and 3. to describe the spatial diversity of CWD strains in North America, enriching our understanding of CWD strain dynamics. By delving into these dimensions, this review sheds light on the intricate interplay among polymorphisms, biochemical properties, and clinical expressions of CWD. This endeavor aims to elevate the trajectory of CWD research, advancing our insight into prion disease.

## 1. Introduction

Prion diseases are fatal transmissible neurodegenerative diseases of mammals; the diseases can have genetic, sporadic, or transmitted etiologies, and include Creutzfeldt-Jakob disease (CJD) in humans, bovine spongiform encephalopathy (BSE) in cattle, scrapie in sheep, and chronic wasting disease (CWD) in cervids [1,2,3,4,5]. The infectious agents do not contain nucleic acids and are composed of misfolded forms of the cellular prion protein, PrP^C^, which is host-encoded by the prion protein gene (*Prnp*) gene [6]. In disease, PrP^C^ is misfolded by template-directed amyloid seeding into abnormal pathogenic isoforms (PrP^Sc^) that cause pathology with distinct disease phenotypes [7,8,9,10]. Disease phenotypes include clinical symptoms, progression rate, survival time, neuropathological alterations, and the biochemical properties of PrP^Sc^ [11,12,13,14]. Importantly, these phenotypes are heritable (i.e., PrP^Sc^ conformations) in the sense they are reproduced faithfully when PrP^Sc^ is transmitted between hosts with similar *Prnp* genetics. These different disease phenotypes are thought to be encoded in the conformation of the PrP^Sc^ and are referred to as prion strains [15]. Strain diversity can explain how prions mutate, evolve, and adapt to novel replication environments such as new host species. How specific conformations of PrP^Sc^ translate to distinct disease phenotypes is still poorly understood [16].

Currently, the conformation of PrP^Sc^ responsible for CWD (hereafter referred to as PrP^CWD^) is the most common animal prion disease [4,5]. We counted more than 17,000 positive cases of CWD that were reported by state and provincial wildlife agencies in 2022 in North America. The disease can transmit vertically and horizontally in both captive and free-ranging cervids. The origin of CWD is unknown with transmission from scrapie-infected sheep, DNA mutation in the *Prnp* gene, or spontaneous misfolding of PrP^C^ all implicated as possible origins [17]. Since first described in the United States in 1967 [18,19], CWD has since been detected in 35 states in the United States, and four provinces in Canada, South Korea, and Scandinavia [20]. CWD strains from Scandinavia are seemingly unique from those in North America [21,22,23,24] and are not reviewed here owing to their recent emergence and ongoing characterization.

The infectious nature of CWD poses a risk to wildlife species and raises concern for its zoonotic potential. Experimental transmission studies using intracranial inoculation demonstrate the proclivity of CWD prions to infect different cervid species [25], hamsters [26,27,28,29,30], bank voles [21,31,32], raccoons [33], sheep [34,35,36], ferrets [37,38], cattle [39,40], laboratory mice [26], and transgenic mouse models expressing a variety of PrP^C^ molecules from different animal species [41], including beaver [42].

Evidence indicates that CWD is propagating as multiple strains [43,44]. Although CWD has been studied for over five decades, knowledge in the diversity and emergence of CWD strains is largely unexplored in terms of distribution and occurrence throughout its geographic range [44,45,46,47]. In some cases, a prion strain may present independently or as a naturally occurring mixture [12,43,48,49,50].

The extensive research conducted on scrapie and BSE has significantly advanced our understanding of prion biology and prion strain properties [4,5,51,52]. Nevertheless, despite efforts by researchers, many questions remain unanswered, such as the origin and the geographic distribution and diversity of the prion strains, which are crucial for understanding prion transmission risk to wildlife and zoonotic potential. The only TSE that has occurred in wild animals is CWD and the number of infected cervids is expected to continue to rise [42]. The objectives of this article are as follows: (1) to address some of the challenges currently faced by researchers studying CWD strains, (2) to provide an overview of the known CWD-infected isolates and their passage history, and (3) to summarize spatial representation of the known CWD strains in North America, including the locations where the affected individual animals were discovered. Due to the unknown source of CWD outside of North America and limited information available for isolate identification, we restrict our review to isolates from North America. By delving into these dimensions, we hope to offer future perspectives on enhancing communication and collaboration among research communities studying CWD strains.

### 1.1. Factors and Challenges in CWD Strain Research

While observing reproducible transmissibility patterns is the most definitive property to identify CWD strains, various factors such as the infectious titer of the CWD isolate, types of tissue, PrP^CWD^ conformation, and the host PrP^C^ primary structure can limit the replication of a prion strain, resulting in extension of already long incubation periods. If replication is too slow, the model host may not be affected within their life span, even if it is infected. Additional passages would be required to characterize the strain by its phenotype. To date, some studies have reported the presence of CWD strains and their unique transmission characteristics (Table 1).

Despite these advances, the diversity of CWD strains circulating in the many CWD enzootic regions remains unknown. The characterization and identification of CWD strains would benefit from inter-laboratory comparisons of transmission properties and phenotypes for particular CWD isolates within the same host models. This is difficult to achieve as only a few laboratories share similar transgenic mouse lines (hereafter referred to as *tg* before a number); however, the usage of standard rodent models (hamsters and non-transgenic mice) could help comparative studies between laboratories. In addition, deposition of transgenic lines to repositories such as the MMRRC program at the Jackson laboratory (https://www.jax.org/research-and-faculty/resources/mutant-mouse-resource-research-center/submitting-mouse-strains, accessed on 25 February 2025) would ensure that they are available for the research community and facilitate inter-laboratory comparison of different CWD isolates.

To date, at least eleven CWD strains have been identified worldwide. Since CWD was first described in 1967, there have been over 50 publications related to CWD strain research, but only 46 infected isolates resulted in the identification of strains or strain mixtures (Table 1). The lack of high throughput methods to screen for strain differences and the limited capacity for confirmatory bioassay studies is limiting the strain characterization of a higher number of CWD cases. Although some publications have acknowledged the importance of using individual isolates in studies [43,45,48,53], there is still a need to expand the reporting of descriptive metadata for isolates including the source, geographic location, and Prnp genotype [47]. In addition, the variety of methods used for strain typing by different labs could be different characterizations of the same strain. Identifying isolates used and potential availability of these isolates may provide samples that are available for a reassessment of strains previously identified to confirm there are five distinct strains in North America.

### 1.2. Strain Typing Data Reproducibility and Comparability

Distinguishing prion strains based solely on sequencing of the *Prnp* gene and phylogenetic analysis is not possible given the limited information this type of approach provides and is not an adequate method for determining transmission properties of prions [42]. While PrP^C^ amino acid polymorphisms do indeed play a critical role in conformational selection, the resulting disease phenotype is a complicated interaction between host and strain [44,66,67,68]. The identification of a specific prion strain requires the use of an extensive panel of assays in vitro and in vivo. These include serial transmission experiments in rodent models, observation of neuropathological patterns in brain regions, and investigation of biochemical and structural properties of PrP^Sc^ conformers [10,42,68,69].

Methods of strain typing are directed to evaluate biochemical properties, such as glycosylation states and migration of proteinase K (PK)-digested products, but also include various assays used to quantify the relative abundance of protease resistant and sensitive PrP^Sc^ such as conformation-dependent immunoassay (CDI) and assays to compare the conformational stability of PrP^Sc^ such as conformational stability assay (CSA), and conformational stability and solubility assay (CSSA), which are particularly useful in differentiation strain-specific conformations [16,63,69]. We have a limited understanding of how the PrP^CWD^ structure encodes strain-specific phenotypes, and how PrP^CWD^ structures emerge and evolve in response to host CWD adaptation.

Research laboratories usually have their own research focus and laboratory-specific protocols, antibodies, and transgenic mouse models. For example, different model organisms and different PrP^C^ expression levels may affect the susceptibility and incubation time of the strain [27,41,70,71]. Transmission studies using different traditional transgenic mice models are difficult to compare between laboratories given potential genomic effects arising from the transgene insertion as well as variable transgene copies inserted (because of random integration of transgene insertion sites), thus constraining comparison between experiments and disease representations [41,71]. Recently, gene-targeted mice expressing species-specific PrP^C^ to several cervid species have emerged as solution to these impediments posed by traditional transgenics; however, these transgenic lines are limited to only a few laboratories (tg; [43,53,72]).

To investigate the interconnections among researchers and determine if certain CWD strain types are being studied by specific research groups, we created social networks based on senior authors (referred to as strain community). We identified seven predominant research groups led by senior authors engaged in collaborative CWD studies involving different species of cervids, transmission studies, genetics of susceptibility, and analysis of PrP^CWD^ biochemical properties in cervids from North America, Europe and South Korea. There is considerable sharing of CWD isolates and strains inside and outside of network, therefore, it would appear beneficial to promote the expansion and formalization of these practices to include other laboratories outside the network as well as the use of a standard panel of commercially available non-transgenic rodents (mice and hamsters) that would allow inter-laboratory comparisons, compliment studies in transgenic mice, and expand the knowledge about transmission properties and zoonotic potential of CWD isolates and strains. The establishment of an inter-laboratory platform or network would provide further insight on the host range of CWD isolates from different regions, help refine the identification of CWD strains by geographic location, and answer whether or not similar strains characterized by different laboratories are different. A formal inter-laboratory network can provide further insight into zoonotic risk and wildlife spill over potential of CWD isolates and strains, as well as reveal their impacts on diagnostics [41,42].

### 1.3. Strain Mixtures in Nature

Current evidence indicates that some CWD natural infections can involve mixtures of strains, such as CWD1/CWD2 and 116AG long/short [43,45]. This phenomenon is not unique to CWD. It has also been observed in humans with sporadic Creutzfeldt–Jakob disease [73,74,75] and in sheep and goats infected with scrapie (Reviewed in [16]). The specific properties of a strain mixture, and how CWD strain diversity is encoded by PrP^Sc^, however, are still unclear. It is uncertain whether the strain mixtures arise from co-existing prion conformers, or if a novel strain evolves under specific conditions by replication of a single conformation species [16,42,75,76,77].

While individual strains have specific PrP^Sc^ conformers with distinct biochemical properties [11,14,50,68,78], it is challenging to detect a novel strain from a strain mixture in any situation. For example, many infected isolates from [78] are strain mixtures of CWD1 and CWD2 (Table 1). Besides unilateral neuropathology and the significantly longer incubation time from CWD2, the two strains have indistinguishable PrP^Sc^ biochemical characteristics that comprise an equivalent ratio of glycoforms, a similar degree of electrophoretic mobility, susceptibility to protease digestion, and similar unfolding characteristics after treatment with guanidinium hydrochloride (Gdn-HCl; [43]).Similarly, two strains were found in the W14-70036 isolate, which contained a mixture of Wisc-1 and a novel strain 116AG [45]. The W14-70036 isolate was characterized by serial passage and phenotyping in tg1536 mice, hamsters and tg60 mice and the resultant phenotypes were compared to passages of a Wisc-1 reference isolate.

In the field, evaluating the strain composition of CWD isolates is challenging due to the unknown passage history. The host range can also play a crucial role in understanding strain selection and adaptation [17,26,42,79,80]. New strains may emerge in populations because of transmission of prions between hosts expressing amino acid polymorphisms in the host PrP^C^. Based on the frequency of non-wildtype alleles in nature, a probable scenario for emergence of novel CWD strains with altered transmission properties is through infection of the cervids expressing PrP^C^ allelic variants that subsequently transmit their novel prions to individuals harboring most common PrP^C^ or other PrP^C^ polymorphisms [48,63]. Passage of CWD from deer exhibiting PrP^C^ polymorphism has been shown to facilitate transmission to species known to resist CWD infection [26]. Transfer of CWD prions to cervids with distinct PrP^C^ molecules could lead to variation in prion conformation, altering transmission characteristics [24,45,48,53,63].

## 2. Materials and Methods

### 2.1. CWD Senior Research Network

**Figure 1:**
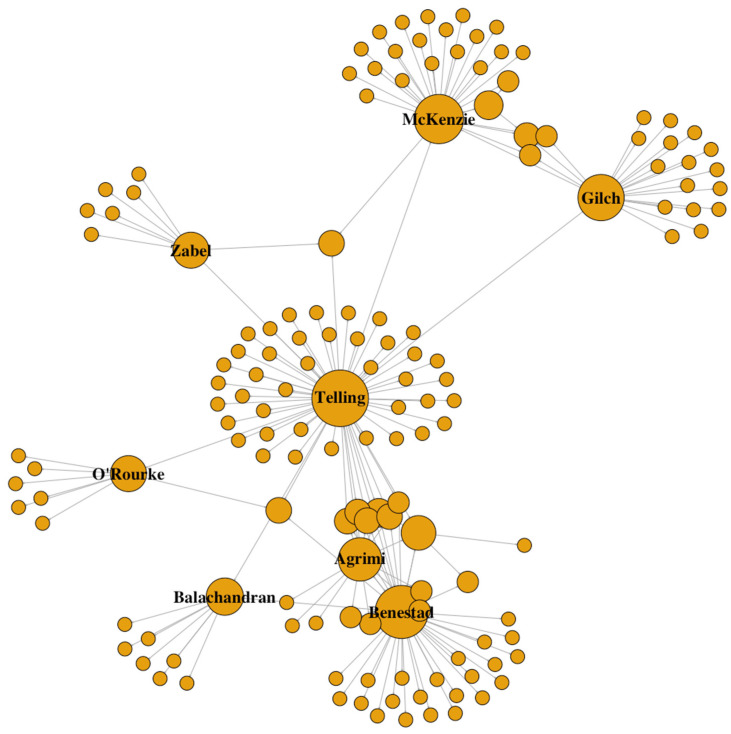
Representation of a social network of chronic wasting disease (CWD) strain researchers showing the connections between senior authors and their collaborators. Each circle represents a researcher. The size of the circle indicates the significance (based on publication numbers and collaboratives) of that researcher in this CWD strain community. The link between the two circles indicates collaboration between the two researchers.

Records of CWD infected isolates were gathered from 140 published articles. Review publications were compiled from PubMed and Center for Infectious Disease Research and Policy (CIDRAP) [81], a website that comprises resources for CWD research. For research published after 2020, we collected from recent publications in CWD newsletter of CIDRAP. We also checked publications of CIDRAP advisory group members and collected publications relevant to CWD strain research. For CWD-infected isolates, we only included the publications that had identified CWD strain type and a recognized isolate source. Therefore, 22 publications and 56 CWD infected isolates met our requirements. To investigate collaboration among researchers/research groups, a research network was constructed based on the senior authors and all additional coauthors in each publication (Figure 1). A total of 93 publications were used in this analysis. To visualize the research network, data were arranged as a social network with each coauthor represented as a “node” and collaborator relationships between senior author and coauthor represented as a “link”. We used net function and igraph package in Program R (v.1.4.3; [82]).

### 2.2. CWD Isolate Review

**Figure 2:**
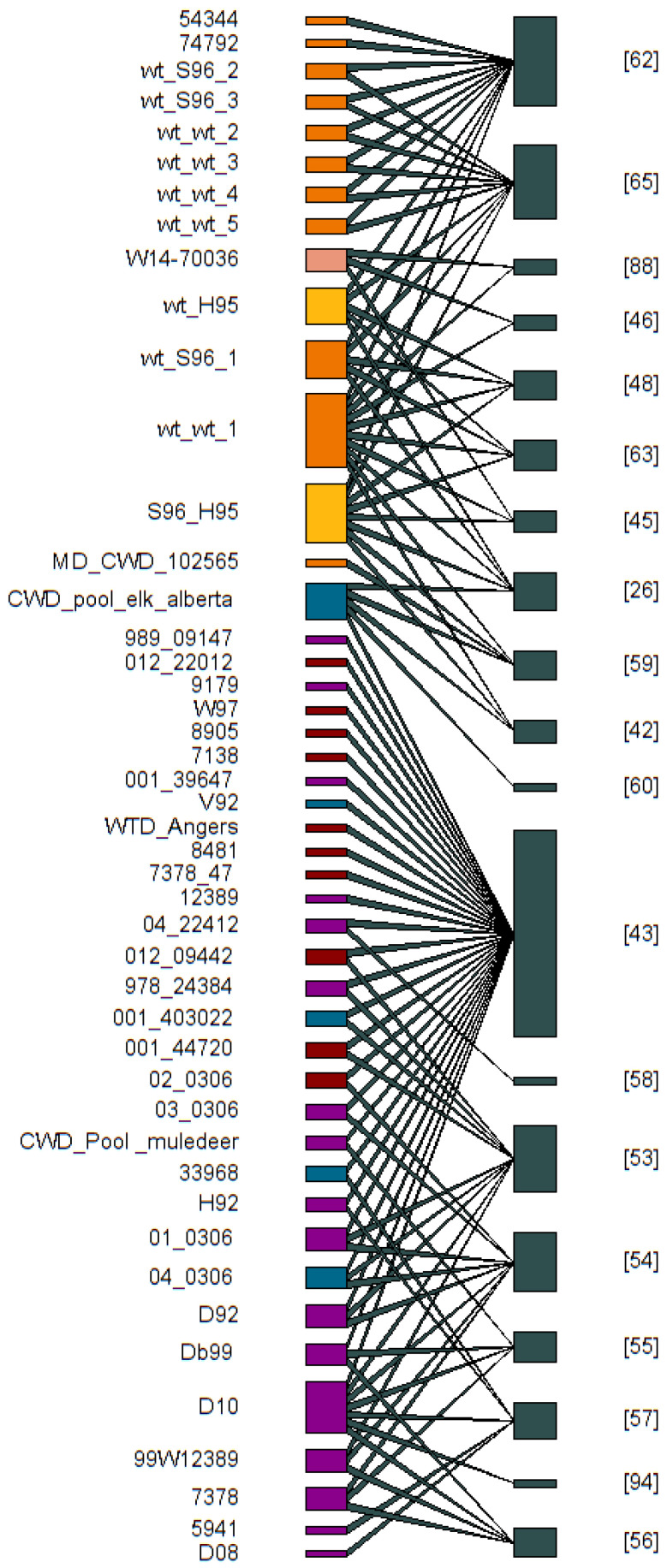
The bipartite network indicates the relationship between infected isolates and their associated publications. The infected isolates column (**left**), width of the colored bar represents the number of publications for that specific infected isolate. The publications column (**right**) represents the number of infected isolates used in the publication.

To visualize the distribution between CWD strain publications and the infected isolate used, we built a bipartite network (Figure 2) by using *plotweb* and *visweb* function in bipartite package in Program R (v2.18; [83]). The lower level indicated the known CWD infected isolates, and the top level indicated the publications that have been applied in that specific isolate. This bipartite representation allowed for identification of the number of times an individual infected isolate had been used in CWD strain research and the number of isolates used in a specific paper.

## 3. Results

### 3.1. North America CWD Strains

To date, eleven potential CWD strains have been described. Here, our focus will be on the five CWD strains documented in free-ranging cervid species in North America, including their infected isolates (Table 1). More information on CWD strains from Nordic countries and captive cervids can be found in a recent review [47].

#### 3.1.1. CWD1 and CWD2

*Infected isolates of CWD1 and CWD2 strains*: Ref. [43] documented CWD1 and CWD2, and their strain mixtures in tg1536 mice. These strains were identified by passage of infected isolates from elk (*Cervus elaphus*) and deer that originated from Colorado, Wyoming, Alberta, and Saskatchewan. These isolates have also served as valuable resources for other research (Table 1).

*Noteworthy information regarding CWD1*: The infected isolate derived from white-tailed deer (*Odocoileus virginianus*), as described by [43], was sourced from Wisconsin and was one of the isolates, which did not result in the CWD2 phenotype during serial passage. This isolate shared similar strain phenotypic features (widespread symmetric neuropathology) with the wt/wt (Wisc-1) infected isolate from Wisconsin described in the work conducted by [48]. Establishing whether Wisc-1 and CWD1 are indeed identical strains remains a challenge, primarily because these prion agents were passaged in different transgenic mice.

*Strain summary*: CWD1 and CWD2 are the strains that were defined from CWD infected isolates from North American cervids [43]. In this study, CWD isolates from elk, mule deer (*Odocoileus hemionus*), and white-tailed deer were inoculated into transgenic mice overexpressing deer PrP^C^ (tg1536mice). The two strains resulted in different disease phenotypes: CWD1 has a shorter incubation time, higher vacuolation score in hippocampus, and a widespread, symmetrical PrP^Sc^ deposition compared to CWD2, which is characterized by longer incubation periods and asymmetrical neuropathology. Transmission of elk isolates typically resulted in either CWD1 or CWD2 with a few contrasting isolates resulting in both phenotypes after first passage (IDs: 12389 and 001-4030022). Of particular interest for comparative studies of CWD strains are the isolates that did not generate mixed phenotypes among the inoculated mouse cohorts (Elk isolate 012-22012 was consistently CWD1 during serial passage). Other isolates with interesting strain instability are those that, upon first passage, generated a single strain phenotype, but in second passage shifted to the other strain phenotype (Isolate 04-0306 was first CWD2 and turned into CWD1 following second passage). This phenotypic shift in strain properties contrasts with isolates that elicited a single phenotype at first passage but during second passage produced the opposite phenotype in a small proportion of the inoculated mouse cohort (i.e., 001-44720).

Serial transmission of mule deer isolates more often resulted in propagation of CWD1 and CWD2 “mixtures” leading to mice with either pathological phenotype [43]. However, various isolates are particularly interesting because their stability during serial passage, including isolate V92 which was consistently the CWD2 strain, and isolates that were mostly CWD1, but which resulted in breakthrough of the CWD2 strain in a small proportion of the inoculated mouse cohorts (IDs: 8481, 978-24384, D10 and Db99 and 001-39647).

The primary structures of mule-deer and elk PrP^C^ differ at codon 226 (Q and E; [84]. Despite this difference, the biochemical properties of PrP^CWD^ such as electrophoretic mobility, conformational stability, and glycoform ratio from both strains were indistinguishable when compared within tg1536 (deer PrP^C^) mice. However, CWD prions propagated in tg1536 mice had different conformation stability than those passaged in tg5037 (elk PrP^C^) after first passage [43].

Perhaps unexpectedly, the CWD1 and CWD2 classification is not phenotypically consistent when the isolates classified by [43] were transmitted in novel gene-targeted transgenic mice expressing deer PrP^C^ [53]. Strain properties can be highly dependent on host factors such as transgene expression level [85]. For example, the Elk isolate 001-44720 was strain-typed as CWD1 (short incubation period in tg1536 [43]) and produced the longest incubation period in GtQ226 mice [53]. In contrast, the transmission of elk isolate 04-0306, which was classified as CWD2 (long incubation period in tg1536), produced relatively shorter incubation periods in GtQ226 mice. These contrasting results may indicate a disconnect between strain specific titers in the cervid isolates and the strain selection by the PrP^C^ expressed in the Gt mice.

Similarly, the PrP^Sc^ deposition pattern, another defining phenotypic feature of CWD1 (symmetric) and CWD2 (asymmetric) is inconsistent when passaged in GtQ226 mice. Transmission of deer or elk isolates classified by [43] as CWD1 (12389) or strain mixtures (D10 and Db99) in GtQ226 produced the asymmetric PrP^CWD^ deposition pattern characteristic of CWD2. In contrast, passage of elk isolates 04-0306 and 12389 (i.e., CWD1) in GtE226 produced symmetric prion aggregates characteristic of CWD1 strain, suggesting the pathology observed arises from strain and host interactions.

#### 3.1.2. Wisc-1/H95^+^

*Infected isolates of Wisc-1 and H95^+^ strain*: The Wisc-1 and H95^+^ strains were characterized from orally inoculated experimental deer [62] and by passage in transgenic mice [48]. These experimental white-tailed deer were comprised of four different groups depending on the PrP^C^ amino acid polymorphisms expressed at codon 95 and 96 (Q95H and G96S). Each deer was inoculated with a brain homogenate pool composed of two CWD positive deer brains (Animal IDs 54344 and 74792). Both animals were hunted in T7N R6E of State Forest section in Iowa County, Wisconsin, where CWD was first identified during the early stage of the outbreak in this state. One deer was a two-year-old female, and the other was a three-year-old female that contained a high abundance of CWD prions in their brain tissue. The Wisc-1 isolates were harvested during November and December 2002. By contrast, the H95^+^ strain is an experimentally derived CWD strain in white-tailed deer and has not been detected in free-ranging deer to date.

The four deer genotypes used to investigate the effect of polymorphisms on incubation time [62] include the following:

1. Q95G96/Q95G96 (wt/wt): Deer expressing *Prnp* genes encoding Q95G96-PrP^C^, also known as wt-PrP^C^, which is canonically the most abundant PrP^C^ molecule in white-tailed deer and mule deer. Deer homozygous for wt-PrP^C^ developed CWD first. Animal IDs: 1293, 1291, 1289, 1277, 1295, and 1287 (preclinical; died of intercurrent gut infection 416 days post-exposure).
2. Q95S96/Q95G96 (S96/wt): Second deer genotype that developed CWD consisted of heterozygous animals expressing wt-PrP^C^ and S96-PrP^C^. Animal IDs: 1281, 1275, and 1285.
3. H95G96/Q95G96 (H95/wt): Third genotype to developed CWD comprised a single heterozygous individual expressing wt-PrP^C^ and H95-PrP^C^. Animal ID: 1279.
4. H95G96/Q95S96 (H95/S96): Last genotype that developed CWD had no wt-PrP^C^ and expressed H95-PrP^C^ and S96-PrP^C^. Animal ID: 1297.

*Strain Summary*: Wisc-1 and 95H+ are two strains that demonstrate the impact of *Prnp* polymorphisms on CWD strain diversification and host range expansion [26,48,63]. To investigate the effect of polymorphisms on incubation time, the above four deer genotypes were used. The data indicate that wt/wt deer exhibited shorter incubation time compared to polymorphic deer and a different protease-resistant was observed in H95/S96 deer (Animal ID 1297; [62].

Transmission studies [48,63] of the CWD proteotypes (Animal IDs 1277, 1293, 1281, 1279 and 1297) described above were conducted in transgenic mice expressing wildtype G96-PrP^C^ (tg33) and S96-PrP^C^ (tg60; [86]). Serial passage in tg33 mice resulted in similar neuropathological, PrP-res profiles and conformational stability indicating a common CWD strain, Wisc-1, was selected. However, most CWD proteotypes failed to produce clinical disease when passed in tg60 mice, except for CWD allotypes (Animal IDs 1279 and 1297), which had H95-PrP^CWD^ and resulted in the identification of a novel CWD strain (H95^+^). The differential strain selection that occurred for CWD allotypes 1279 and 1297, indicates PrP^C^ heterozygous deer accumulated a strain mixture (Wisc-1 and H95^+^), and the strain selection was dependent on PrP^C^ of the inoculated tg mouse line, with tg33 selecting the Wisc-1 strain while tg60 selected H95^+^. Importantly, passage of tg60-CWD-H95^+^ into tg33 mice resulted in some mice displaying the Wisc-1 phenotype while others displayed the H95^+^ phenotype, demonstrating the H95^+^ strain can also be propagated in host expressing wt-PrP^C^ [48]. Likewise, the transmission of tg33 prions into tg60 mice resulted in H95^+^ selection when the donor tg33 mice received the 1297 or 1279 CWD allotypes [63]. These results indicate that H95^+^ strain emerged in deer expressing H95-PrP^C^ as a result of conformational diversification as opposed to selection from the original CWD pool used to infect the polymorphic deer.

To further investigate host range of the two strains, ref. [26] inoculated non-transgenic C57BI6 mice with isolates (Animal IDs 1293, 1281, 1279 and 1297). Surprisingly, the results showed that only mice infected with isolates from animal IDs 1279 and 1297 (containing the H95^+^ strain) produced clinical disease in C57BI6 mice. This finding also suggests the unique transmission properties of the H95^+^ strain, as it can propagate in a wider range of host species.

The experimental CWD isolates also exhibited PrP^Sc^ biochemical and structural differences when heterozygous CWD isolates were compared to wt/wt homozygous deer prions [63]. The PrP^CWD^ of the H95^+^ strain has greater structural stability than the Wisc-1 strain and tg33 mice exposed to H95/S96 CWD prions (i.e.,1297) contain sub-populations of PrP^CWD^ that were also more stable than those from tg33 mice exposed to 1293 [63]. Moreover, despite isolates from animal IDs 1293, 1277 and 1281 not causing prion disease in tg60 mice, they managed to establish a subclinical infection in mice after more than 600 days post infection with correspondingly low levels of PrP-res in their brains. Subclinically infected mice contained approximately 1/10 of the PrP-res accumulated in tg60 that succumbed to H95^+^ [63]. Analysis of the allelotype composition of PrP^CWD^ in wt/wt and S96/wt isolates demonstrated that wt-PrP^C^ is more susceptible to CWD prion misfolding than S96-PrP^C^ and converted with different efficiencies in S96/wt deer [87]. Findings from serial in vitro protein misfolding cyclic amplification (PMCA) experiments also demonstrated that S96-PrP^C^ leads to inefficient in vitro propagation of PrP^CWD^ for most tested strains, with H95^+^ being able to propagate continually [59].

#### 3.1.3. 116AG

*Infected isolate of 116AG strain*: The infected isolate 116AG originated from a five-year-old male white-tailed deer with original animal ID W14-70036. The deer was free-ranging and found as roadkill in Saskatchewan province. Since it was a wild animal, there is no available information about the passage history of the isolate. The deer appeared emaciated, and it tested positive for CWD using immunohistochemistry. The infected isolate was from a deer heterozygous for PrPs [45].

*Strain Summary*: The strain 116AG is an example of how polymorphisms at codon 116 (A to G) in PrP^C^ of white-tailed deer can impact the stability, pathogenesis and prion properties of CWD [45]. The PrP^Sc^ from infected isolate of heterozygous PrP^C^ deer (116AG) was conformationally less stable than PrP^Sc^ from the experimental Wisc-1 isolate (Animal ID: 1277) from PrP^C^ wt/wt deer [64]. As compared to Wisc-1, PrP^Sc^ from isolate 116AG shows lower seeding activity in real-time quaking-induced conversion (RT-QuIC), slower disease propagation, longer incubation time in different rodent models and reduced infectivity in vitro [45,88]. Serial transmission in tg(CerPrP)1536^+/−^ revealed the infected isolate, W14-70036 contained two strains, 116AG-long and 116AG-short. In the third passage, 116AG-long displayed a prolonged two-month clinical phase delay, prolonged survival and disease progression. The proteinase K-resistant banding patterns of PrP^Sc^ from the two strains also displayed distinctive electrophoretic mobility in both tg(CerPrP)1536^+/−^ mice and Syrian golden hamsters [45].

#### 3.1.4. 132LL Elk CWD

Elk are polymorphic at residue 132 of the prion protein and the presence of a leucine or methionine has significant disease modifying effects that suggest novel strain emergence [89,90]. The incubation period of CWD was extended in elk with 132LL > 132ML > 132MM. PrP^CWD^ was less abundant in clinically affected 132LL elk as compared to clinically affected 132MM elk. 132LL elk CWD prions were differentially cleaved by proteinase-K to a lower molecular weight and had increased structural stability as compared to 132MM CWD prions. These biochemical alterations indicate novel strain emergence in isolates from 132LL elk. Subsequent experimental transmission of 132LL CWD Prions into Tg12 (132MM) transgenic mice [91] supports strain emergence [92]. When the 132LL CWD prions were passaged, they maintained their distinctive biochemical phenotypes including increased conformational stability and low molecular weight proteinase-K cleave PrP-res isoforms.

#### 3.1.5. Other Isolates of CWD in North America

The data demonstrating that an isolate is composed of a novel strain of CWD typically include biochemical, transmission and pathology data that show a heritable phenotype upon passage. Other isolates of CWD that are likely composed of novel strains include CWD in mule deer with the 225FF polymorphism [93,94] as well as moose (*Alces alces*) CWD [24,95].

### 3.2. Spatial Representation of North American CWD Strains

Two paths can conceivably contribute to the geographic distribution of CWD; one is from captive cervids, and the other is spread among free-ranging cervids. Before 2000, the distribution of CWD in free-ranging cervids has only been reported in Colorado and Wyoming. Subsequently, CWD was concentrated in four main enzootic zones: 1. northeastern Colorado and southeastern Wyoming, 2. Saskatchewan, Canada, 3. Wisconsin, and 4. Mid-Atlantic [20]. The spread of CWD to neighboring states/regions could have originated from these, with subsequent jumps leading to the establishment of new foci. Regardless of how CWD spreads remains controversial, with limited documented evidence necessitating further research [96].

**Figure 3:**
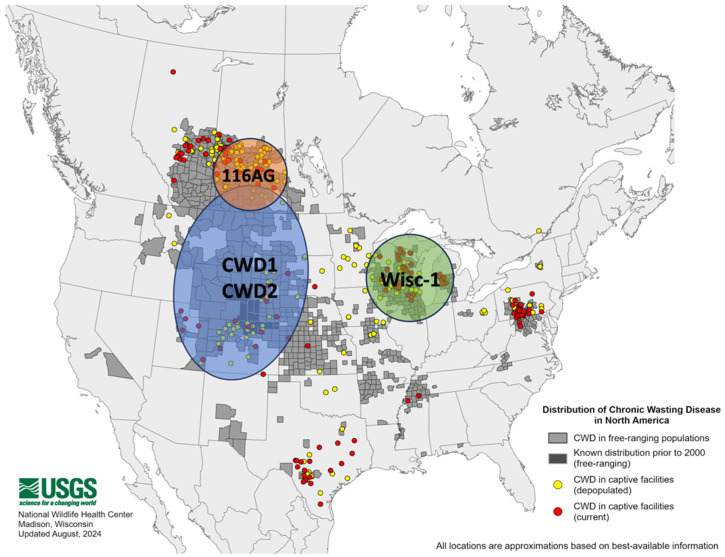
Geographic distribution of predicted and known chronic wasting disease (CWD) strains and their respective endemic zone in free-ranging cervids in North America. Chronic wasting disease endemic zones translated from within the remaining range of positive cervids currently unknown as to strain type [20].

To advance our understanding of CWD strains, having well-documented records of infected isolates is imperative. Historical records of infected isolates (Table 1) suggest that several enzootic zones of CWD appear to represent the spatial distribution of known strains in North America based on a few animals that have had strain explored (Figure 3). A potential distribution of CWD 1 and CWD2 could be found in mule deer and elk in Colorado and Wyoming, United States, and Saskatchewan, Canada, while Wisc-1 was detected in white-tailed deer in Wisconsin, United States. The 116AG was isolated from Saskatchewan, Canada, although this is likely a rare event. The spatial distribution of cervid species may also play an essential role in the distribution of CWD strains. Interaction between cervid species and horizontal transmission may promote strain mixing.

Importantly, whether the enzootic zones are the extension of the origin strain region, a secondary region, or a separate, spontaneous emergence of the CWD strain is difficult to identify. The emergence of novel CWD strains is transmitted through animal populations. Given that geographic variation in *Prnp* can impact susceptibility to prion disease, this can also result in the development of distinct strain characteristics. For instance, an investigation on human *Prnp* from major continental groups affected by CJD found significant geographic variation in susceptibility based on allele frequency [97]. Notably, the 129V allele is highly represented in certain populations in the United States, which predicted that Native Americans would be less susceptible to prion infection than other populations due to a higher rate of heterozygotes M/V. Heterozygotes are associated with a lower probability of contracting CJD whereas the protective 219K allele, which lowers the risk of CJD, was constantly linked with 129M and primarily confined to Asian and Pacific populations but less so in the Native American population. The counteracting susceptibility at codon 129 may lower the CJD contracting rate in those regions [97]. Therefore, polymorphism of *Prnp* gene among geographically varied cervid populations should be considered when modeling the epidemiological properties of CWD, as the generation of new CWD strain can be transmitted through a population and result in novel disease characteristics [47].

The origin of CWD and the most prevalent CWD strain present in nature remains elusive, and information about the epidemiology of the CWD strain is often limited. In scrapie, multiple transmission experiments in laboratories, from sheep to mice, have resulted in the same strain, ME7 [98,99,100]. Furthermore, natural scrapie transmission to mice from 26 geographic resources around the United Kingdom found that over half of the mice developed clinical signs similar to the ME7 strain [100]. If we could obtain CWD-infected isolates from free-ranging cervids from diverse geographic regions and compare their strain properties when transmitted to a panel of transgenic mice, more reliable evidence regarding the distribution of CWD strains would be available. Additionally, by comparing the properties of CWD strains between newly emergent and endemic locations, we may identify the epidemiological origin of CWD in new locations [41]. Comparable work with sheep and goats has been useful to define the temporal and geographical distribution of scrapie [98,99,101,102,103].

## 4. Discussion

In our current effort, we have attempted to address some current challenges facing CWD strain research. While decades of prion research have provided a much deeper understanding of the mechanical framework of prion strains, many questions remain unanswered and unexplored in CWD. For example, little is known about the origin of strains and the mechanisms that drive the diversification and expansion of CWD strains into different host species. Additionally, we are unsure how host species manage to coexist with a mixture of strains and support multiple strains simultaneously. An extensive collection of infected isolates from across geographic regions appears warranted to answer these vital and unrequited questions. In recent years, there has been an increased focus on CWD surveillance, and the infected isolates used for CWD testing that could be made available for genotyping, strain typing, and transmission studies [44]. In 2019, a large contingent of researchers studying chronic wasting disease was formed, prioritizing establishing a tissue repository to increase the accessibility of CWD isolates [104].

Recent studies have shown that Nordic cervids carry a variety of strain profiles, contrasting with a relatively constant strain profile in North American cervids [22]. Arguments can be made, however, that the diversity of North American CWD strains is still underestimated. A more connected research network would enable tracking of CWD strains and genotypes. As CWD strains evolve [48,63], new CWD strains can emerge with unpredictable disease phenotypes and host ranges [26,42,80]. Because of the increasing prevalence of CWD, and therefore, increased exposure to CWD, there is a growing concern about transmission to humans and other species [4,46,51,105].

As the emergence of novel strains increases the zoonotic potential, developing and standardizing methods to efficiently identify new strains and their transmission is warranted to document CWD spread [41]. New approaches include amplification-based assays such as PMCA are offering the possibility to distinguish CWD strains. Forty-five CWD+ retropharyngeal lymph node samples from Texas were screened and identified a single PMCA amplified isolate (64420) migrated faster on Western blot [106]. In another example, PMCA was used with five different substrates to amplify CWD+ isolates from six cervid species [23]. One of the isolates, DB99 (Table 1), was previously identified as a mixture of CWD1 and CWD2. Further characterization of isolates by PMCA may enhance confidence in their distinctiveness or similarity. Other exciting work for understanding CWD strains includes the first cryo-EM images and structural analysis of CWD [107]. It seems likely that structures for other CWD strains will soon be completed. Understanding the structures that encode the different strains of CWD is particularly important to explain the disease modifying properties of different polymorphisms. It is unclear how the G96S polymorphism can extend incubation period in Wisc-1/CWD1 infected deer or how the Q95H can drive the emergence of H95^+^. Structural information that explains this would increase our understanding of prion replication.

## Figures and Tables

**Table 1:** Infected isolates of chronic wasting disease (CWD) strains by animal identification (Animal ID), strain identified, species, state or province (state), tissue, and reference cited.

Animal_ID	Strain	Species	State	Tissue	References
012-09442	CWD1	Elk	Colorado	Brain	[43,53]
012-22012	CWD1	Elk	Colorado	Brain	[43]
001-44720	CWD1/CWD2	Elk	Colorado	Brain	[43,53]
02-0306	CWD1/CWD2	Elk	Saskatchewan	Antler velvet, Brain	[43,54]
7378-47	CWD1/CWD2	Elk	Wyoming	Brain	[43,54,55,56]
W97	CWD1/CWD2	Mule Deer	Colorado	Brain	[43]
7138	CWD1/CWD2	Mule Deer	Wyoming	Brain	[43]
8481	CWD1/CWD2	Mule Deer	Wyoming	Brain	[43]
8905	CWD1/CWD2	Mule Deer	Wyoming	Brain	[43]
WTD_Angers	CWD1	White-tailed Deer	Wisconsin	Brain	[43]
01-0306	CWD2	Elk	Saskatchewan	Antler velvet, Brain	[43,53,54]
03-0306	CWD2	Elk	Saskatchewan	Antler velvet, Brain	[43,54]
99W12389	CWD1/CWD2	Elk	Wyoming	Brain	[43,53,54,56]
001-39647	CWD1/CWD2	Mule Deer	Colorado	Brain	[43]
989-09147	CWD1/CWD2	Mule Deer	Colorado	Brain	[43]
D08	CWD1/CWD2	Mule Deer	Colorado	Muscle	[57]
H92	CWD1/CWD2	Mule Deer	Colorado	Brain, Muscle	[43,57]
CWD Pool	CWD1/CWD2	Mule Deer	Colorado (captive)	Brain	[43]
D10	CWD1/CWD2	Mule Deer	Colorado (captive)	Brain, Muscle	[43,53,54,55,56,57]
D92	CWD1/CWD2	Mule Deer	Colorado (captive)	Brain	[43,54]
Db99	CWD1/CWD2	Mule Deer	Colorado (captive)	Brain	[43,53,55,56]
978-24384	CWD1/CWD2	Mule Deer	Colorado	Brain	[43,53]
9179	CWD1/CWD2	Mule Deer	Wyoming	Brain	[43]
04-22412	CWD1/CWD2	Mule Deer	Wyoming	Brain	[43,58]
5941	CWD1/CWD2	Mule Deer	Colorado	Muscle	[57]
CWD_Elk pool	CWD2	Elk	Alberta (captive)	Brain	[26,42,43,59,60,61]
001-403022	CWD2	Elk	Colorado	Brain	[43,53]
04-0306	CWD2	Elk	Saskatchewan	Antler velvet, Brain	[43,53,54]
V92	CWD2	Mule Deer	Colorado	Brain	[43]
33968	CWD2	Mule Deer	Colorado	Brain, Muscle	[43,57]
1275 (96GS)	Wisc-1	White-tailed Deer	Wisconsin	Brain	[62]
1277 (96GG)	Wisc-1	White-tailed Deer	Wisconsin	Brain	[48,62,63]
1279 (95QH/96GS)	H95^+^/Wisc-1	White-tailed Deer	Wisconsin	Brain	[26,45,48,59,62,63]
1281 (96GS)	Wisc-1	White-tailed Deer	Wisconsin	Brain	[26,45,48,59,62,63,64,65]
1285 (96GS)	Wisc-1	White-tailed Deer	Wisconsin	Brain	[62]
1289 (96GG)	Wisc-1	White-tailed Deer	Wisconsin	Brain	[62]
1291 (96GG)	Wisc-1	White-tailed Deer	Wisconsin	Brain	[62]
1293 (96GG)	Wisc-1	White-tailed Deer	Wisconsin	Brain	[26,45,48,59,62,63,64]
1295 (96GG)	Wisc-1	White-tailed Deer	Wisconsin	Brain	[62]
1297 (95QH/96GG)	Wisc-1/H95^+^	White-tailed Deer	Wisconsin	Brain	[26,44,45,48,59,62,63]
54344 (96GG)	Wisc-1	White-tailed Deer	Wisconsin	Brain	[62]
74792 (96GG)	Wisc-1	White-tailed Deer	Wisconsin	Brain	[62]
W14-70036	116AG	White-tailed Deer	Saskatchewan	Brain	[45,64]

## Abbreviations

The following abbreviations are used in this manuscript:

CWD	Chronic wasting disease
PMCA	Protein misfolding cyclic amplification
PrP^C^	Normal prion protein
Prnp	Prion protein gene
CJD	Creutzfeldt–Jakob disease

## References

[1] Béringue V., Vilotte J.L., Laude H. (2008). Prion Agent Diversity and Species Barrier. Vet. Res..

[2] Daus M.L., Breyer J., Wagenfuehr K., Wemheuer W.M., Thomzig A., Schulz-Schaeffer W.J., Beekes M. (2011). Presence and Seeding Activity of Pathological Prion Protein (PrP(TSE)) in Skeletal Muscles of White-Tailed Deer Infected with Chronic Wasting Disease. PLoS ONE.

[3] Daus M.L., Beekes M. (2012). Chronic Wasting Disease: Fingerprinting the Culprit in Risk Assessments. Prion.

[4] Houston F., Andréoletti O. (2019). Animal Prion Diseases: The Risks to Human Health. Brain Pathol..

[5] Imran M., Mahmood S. (2011). An Overview of Animal Prion Diseases. Virol. J..

[6] Oesch B., Westaway D., Wälchli M., McKinley M.P., Kent S.B., Aebersold R., Barry R.A., Tempst P., Teplow D.B., Hood L.E. (1985). A Cellular Gene Encodes Scrapie PrP 27-30 Protein. Cell.

[7] Race R.E., Raines A., Baron T.G., Miller M.W., Jenny A., Williams E.S. (2002). Comparison of Abnormal Prion Protein Glycoform Patterns from Transmissible Spongiform Encephalopathy Agent-Infected Deer, Elk, Sheep, and Cattle. J. Virol..

[8] Race B., Meade-White K.D., Miller M.W., Barbian K.D., Rubenstein R., LaFauci G., Cervenakova L., Favara C., Gardner D., Long D. (2009). Susceptibilities of Nonhuman Primates to Chronic Wasting Disease. Emerg. Infect. Dis..

[9] Johnson C.J., Aiken J.M., McKenzie D., Samuel M.D., Pedersen J.A. (2012). Highly Efficient Amplification of Chronic Wasting Disease Agent by Protein Misfolding Cyclic Amplification with Beads (PMCAb). PLoS ONE.

[10] Meyerett-Reid C., Wyckoff A.C., Spraker T., Pulford B., Bender H., Zabel M.D. (2017). *De Novo* Generation of a Unique Cervid Prion Strain Using Protein Misfolding Cyclic Amplification. mSphere.

[11] Aguzzi A., Heikenwalder M., Polymenidou M. (2007). Insights into Prion Strains and Neurotoxicity. Nat. Rev. Mol. Cell Biol..

[12] Collinge J. (2010). Prion Strain Mutation and Selection. Science.

[13] Holec S.A.M., Yuan Q., Bartz J.C. (2019). Alteration of Prion Strain Emergence by Nonhost Factors. mSphere.

[14] Telling G.C. (2010). Nucleic Acid-Free Mutation of Prion Strains. Prion.

[15] Bessen R.A., Kocisko D.A., Raymond G.J., Nandan S., Lansbury P.T., Caughey B. (1995). Non-Genetic Propagation of Strain-Specific Properties of Scrapie Prion Protein. Nature.

[16] Block A.J., Bartz J.C. (2023). Prion Strains: Shining New Light on Old Concepts. Cell Tissue Res..

[17] Pritzkow S. (2022). Transmission, Strain Diversity, and Zoonotic Potential of Chronic Wasting Disease. Viruses.

[18] Williams E.S., Young S. (1980). Chronic Wasting Disease of Captive Mule Deer: A Spongiform Encephalopathy. J. Wildl. Dis..

[19] Williams E.S., Young S. (1982). Spongiform Encephalopathy of Rocky Mountain Elk. J. Wildl. Dis..

[20] [USGS], United States Geological Survey. Expanding Distribution of Chronic Wasting Disease. United States Geological Survey [USGS]. https://www.usgs.gov/centers/nwhc/science/expanding-distribution-chronic-wasting-disease?qt-science_center_objects=0#qt-science_center_objects.

[21] Nonno R., Di Bari M.A., Pirisinu L., D’Agostino C., Vanni I., Chiappini B., Marcon S., Riccardi G., Tran L., Vikøren T. (2020). Studies in Bank Voles Reveal Strain Differences between Chronic Wasting Disease Prions from Norway and North America. Proc. Natl. Acad. Sci. USA.

[22] Sun J.L., Kim S., Crowell J., Webster B.K., Raisley E.K., Lowe D.C., Bian J., Korpenfelt S.L., Benestad S.L., Telling G.C. (2023). Novel Prion Strain as Cause of Chronic Wasting Disease in a Moose, Finland. Emerg. Infect. Dis..

[23] Pritzkow S., Gorski D., Ramirez F., Telling G.C., Benestad S.L., Soto C. (2021). North American and Norwegian Chronic Wasting Disease Prions Exhibit Different Potential for Interspecies Transmission and Zoonotic Risk. J. Infect. Dis..

[24] Bian J., Kim S., Kane S.J., Crowell J., Sun J.L., Christiansen J., Saijo E., Moreno J.A., DiLisio J., Burnett E. (2021). Adaptive Selection of a Prion Strain Conformer Corresponding to Established North American CWD During Propagation of Novel Emergent Norwegian Strains in Mice Expressing Elk or Deer Prion Protein. PLoS Pathog..

[25] Hamir A.N., Greenlee J.J., Nicholson E.M., Kunkle R.A., Richt J.A., Miller J.M., Hall M. (2011). Experimental Transmission of Chronic Wasting Disease (CWD) from Elk and White-Tailed Deer to Fallow Deer by Intracerebral Route: Final Report. Can. J. Vet. Res..

[26] Herbst A., Velásquez C.D., Triscott E., Aiken J.M., McKenzie D. (2017). Chronic Wasting Disease Prion Strain Emergence and Host Range Expansion. Emerg. Infect. Dis..

[27] Raymond G.J., Raymond L.D., Meade-White K.D., Hughson A.G., Favara C., Gardner D., Williams E.S., Miller M.W., Race R.E., Caughey B. (2007). Transmission and Adaptation of Chronic Wasting Disease to Hamsters and Transgenic Mice: Evidence for Strains. J. Virol..

[28] Bessen R.A., Robinson C.J., Seelig D.M., Watschke C.P., Lowe D., Shearin H., Martinka S., Babcock A.M. (2011). Transmission of Chronic Wasting Disease Identifies a Prion Strain Causing Cachexia and Heart Infection in Hamsters. PLoS ONE.

[29] Crowell J., Hughson A., Caughey B., Bessen R.A. (2015). Host Determinants of Prion Strain Diversity Independent of Prion Protein Genotype. J. Virol..

[30] Baral P.K., Swayampakula M., Aguzzi A., James M.N. (2015). X-ray Structural and Molecular Dynamical Studies of the Globular Domains of Cow, Deer, Elk and Syrian Hamster Prion Proteins. J. Struct. Biol..

[31] Orrú C.D., Groveman B.R., Raymond L.D., Hughson A.G., Nonno R., Zou W., Ghetti B., Gambetti P., Caughey B. (2015). Bank Vole Prion Protein as an Apparently Universal Substrate for RT-QuIC-Based Detection and Discrimination of Prion Strains. PLoS Pathog..

[32] Watts J.C., Giles K., Patel S., Oehler A., DeArmond S.J., Prusiner S.B. (2014). Evidence That Bank Vole PrP Is a Universal Acceptor for Prions. PLoS Pathog..

[33] Moore S.J., Carlson C.M., Schneider J.R., Johnson C.J., Greenlee J.J. (2022). Increased Attack Rates and Decreased Incubation Periods in Raccoons with Chronic Wasting Disease Passaged Through Meadow Voles. Emerg. Infect. Dis..

[34] Cassmann E.D., Moore S.J., Greenlee J.J. (2021). Experimental Oronasal Transmission of Chronic Wasting Disease Agent from White-Tailed Deer to Suffolk Sheep. Emerg. Infect. Dis..

[35] Cassmann E.D., Frese R.D., Greenlee J.J. (2021). Second Passage of Chronic Wasting Disease of Mule Deer to Sheep by Intracranial Inoculation Compared to Classical Scrapie. J. Vet. Diagn. Investig..

[36] Harrathi C., Fernández-Borges N., Eraña H., Elezgarai S.R., Venegas V., Charco J.M., Castilla J. (2019). Insights into the Bidirectional Properties of the Sheep-Deer Prion Transmission Barrier. Mol. Neurobiol..

[37] Bartz J.C., Marsh R.F., McKenzie D.I., Aiken J.M. (1998). The Host Range of Chronic Wasting Disease Is Altered on Passage in Ferrets. Virology.

[38] Perrott M.R., Sigurdson C.J., Mason G.L., Hoover E.A. (2012). Evidence for Distinct Chronic Wasting Disease (CWD) Strains in Experimental CWD in Ferrets. J. Gen. Virol..

[39] Greenlee J.J., Nicholson E.M., Smith J.D., Kunkle R.A., Hamir A.N. (2012). Susceptibility of Cattle to the Agent of Chronic Wasting Disease from Elk after Intracranial Inoculation. J. Vet. Diagn. Investig..

[40] Raymond G.J., Bossers A., Raymond L.D., O’Rourke K.I., McHolland L.E., Bryant P.K., Miller M.W., Williams E.S., Smits M., Caughey B. (2000). Evidence of a Molecular Barrier Limiting Susceptibility of Humans, Cattle and Sheep to Chronic Wasting Disease. EMBO J..

[41] Cook M., Hensley-McBain T., Grindeland A. (2023). Mouse Models of Chronic Wasting Disease: A Review. Front. Virol..

[42] Herbst A., Wohlgemuth S., Yang J., Castle A.R., Moreno D.M., Otero A., Aiken J.M., Westaway D., McKenzie D. (2022). Susceptibility of Beavers to Chronic Wasting Disease. Biology.

[43] Angers R.C., Kang H.E., Napier D., Browning S., Seward T., Mathiason C., Balachandran A., McKenzie D., Castilla J., Soto C. (2010). Prion Strain Mutation Determined by Prion Protein Conformational Compatibility and Primary Structure. Science.

[44] Arifin M.I., Hannaoui S., Chang S.C., Thapa S., Schatzl H.M., Gilch S. (2021). Cervid Prion Protein Polymorphisms: Role in Chronic Wasting Disease Pathogenesis. Int. J. Mol. Sci..

[45] Hannaoui S., Triscott E., Duque Velásquez C., Chang S.C., Arifin M.I., Zemlyankina I., Tang X., Bollinger T., Wille H., McKenzie D. (2021). New and Distinct Chronic Wasting Disease Strains Associated with Cervid Polymorphism at Codon 116 of the Prnp Gene. PLoS Pathog..

[46] Hannaoui S., Zemlyankina I., Chang S.C., Arifin M.I., Béringue V., McKenzie D., Schatzl H.M., Gilch S. (2022). Transmission of Cervid Prions to Humanized Mice Demonstrates the Zoonotic Potential of CWD. Acta Neuropathol..

[47] Otero A., Duque Velasquez C., McKenzie D., Aiken J. (2023). Emergence of CWD Strains. Cell Tissue Res..

[48] Duque Velásquez C., Kim C., Herbst A., Daude N., Garza M.C., Wille H., Aiken J., McKenzie D. (2015). Deer Prion Proteins Modulate the Emergence and Adaptation of Chronic Wasting Disease Strains. J. Virol..

[49] Béringue V., Herzog L., Jaumain E., Reine F., Sibille P., Le Dur A., Vilotte J.L., Laude H. (2012). Facilitated Cross-Species Transmission of Prions in Extraneural tissue. Science.

[50] Collinge J., Clarke A.R. (2007). A General Model of Prion Strains and Their Pathogenicity. Science.

[51] Bosque P.J. (2002). Bovine Spongiform Encephalopathy, Chronic Wasting Disease, Scrapie, and the Threat to Humans from Prion Disease Epizootics. Curr. Neurol. Neurosci. Rep..

[52] Bruce M.E., Boyle A., Cousens S., McConnell I., Foster J., Goldmann W., Fraser H. (2002). Strain Characterization of Natural Sheep Scrapie and Comparison with BSE. J. Gen. Virol..

[53] Bian J., Christiansen J.R., Moreno J.A., Kane S.J., Khaychuk V., Gallegos J., Kim S., Telling G.C. (2019). Primary Structural Differences At Residue 226 of Deer and Elk PrP Dictate Selection of Distinct CWD Prion Strains in Gene-Targeted Mice. Proc. Natl. Acad. Sci. USA.

[54] Angers R.C., Seward T.S., Napier D., Green M., Hoover E., Spraker T., O’Rourke K., Balachandran A., Telling G.C. (2009). Chronic Wasting Disease Prions in Elk Antler Velvet. Emerg. Infect. Dis..

[55] Browning S.R., Mason G.L., Seward T., Green M., Eliason G.A., Mathiason C., Miller M.W., Williams E.S., Hoover E., Telling G.C. (2004). Transmission of Prions from Mule Deer and Elk with Chronic Wasting Disease to Transgenic Mice Expressing Cervid PrP. J. Virol..

[56] Green K.M., Browning S.R., Seward T.S., Jewell J.E., Ross D.L., Green M.A., Williams E.S., Hoover E.A., Telling G.C. (2008). The Elk PRNP Codon 132 Polymorphism Controls Cervid and Scrapie Prion Propagation. J. Gen. Virol..

[57] Angers R.C., Browning S.R., Seward T.S., Sigurdson C.J., Miller M.W., Hoover E.A., Telling G.C. (2006). Prions in Skeletal Muscles of Deer with Chronic Wasting Disease. Science.

[58] Green K.M., Castilla J., Seward T.S., Napier D.L., Jewell J.E., Soto C., Telling G.C. (2008). Accelerated High Fidelity Prion Amplification within and Across Prion Species Barriers. PLoS Pathog..

[59] Otero A., Duque Velásquez C., Aiken J., McKenzie D. (2021). White-Tailed Deer S96 Prion Protein Does Not Support Stable In Vitro Propagation of Most Common CWD Strains. Sci. Rep..

[60] Pushie M.J., Shaykhutdinov R., Nazyrova A., Graham C., Vogel H.J. (2011). An NMR Metabolomics Study of Elk Inoculated with Chronic Wasting Disease. J. Toxicol. Environ. Health A.

[61] Gordon P.M., Schütz E., Beck J., Urnovitz H.B., Graham C., Clark R., Dudas S., Czub S., Sensen M., Brenig B. (2009). Disease-specific Motifs Can Be Identified in Circulating Nucleic Acids from Live Elk and Cattle Infected with Transmissible Spongiform Encephalopathies. Nucleic Acids Res..

[62] Johnson C.J., Herbst A., Duque-Velasquez C., Vanderloo J.P., Bochsler P., Chappell R., McKenzie D. (2011). Prion Protein Polymorphisms Affect Chronic Wasting Disease Progression. PLoS ONE.

[63] Duque Velásquez C., Kim C., Haldiman T., Herbst A., Aiken J., Safar J.G., McKenzie D. (2020). Chronic Wasting Disease (CWD) Prion Strains Evolve via Adaptive Diversification of Conformers in Hosts Expressing Prion Protein Polymorphisms. J. Biol. Chem..

[64] Hannaoui S., Amidian S., Cheng Y.C., Duque Velásquez C., Dorosh L., Law S., Telling G., Stepanova M., McKenzie D., Wille H. (2017). Destabilizing Polymorphism in Cervid Prion Protein Hydrophobic Core Determines Prion Conformation and Conversion Efficiency. PLoS Pathog..

[65] Otero A., Duque Velásquez C., Johnson C., Herbst A., Bolea R., Badiola J.J., Aiken J., McKenzie D. (2019). Prion Protein Polymorphisms Associated with Reduced CWD Susceptibility Limit Peripheral PrP. BMC Vet. Res..

[66] Dickinson A.G., Meikle V.M. (1971). Host-Genotype and Agent Effects in Scrapie Incubation: Change in Allelic Interaction with Different Strains of Agent. Mol. Gen. Genet..

[67] Cortez L.M., Sim V.L. (2013). Implications of Prion Polymorphisms. Prion.

[68] Carta M., Aguzzi A. (2022). Molecular Foundations of Prion Strain Diversity. Curr. Opin. Neurobiol..

[69] Safar J.G., Xiao X., Kabir M.E., Chen S., Kim C., Haldiman T., Cohen Y., Chen W., Cohen M.L., Surewicz W.K. (2015). Structural Determinants of Phenotypic Diversity and Replication Rate of Human Prions. PLoS Pathog..

[70] Tamgüney G., Giles K., Bouzamondo-Bernstein E., Bosque P.J., Miller M.W., Safar J., DeArmond S.J., Prusiner S.B. (2006). Transmission of Elk and Deer Prions to Transgenic Mice. J. Virol..

[71] Watts J.C., Prusiner S.B. (2014). Mouse Models for Studying the Formation and Propagation of Prions. J. Biol. Chem..

[72] Arifin M.I., Hannaoui S., Ng R.A., Zeng D., Zemlyankina I., Ahmed-Hassan H., Schatzl H.M., Kaczmarczyk L., Jackson W.S., Benestad S.L. (2024). Norwegian Moose CWD Induces Clinical Disease and Neuroinvasion in Gene-Targeted Mice Expressing Cervid S138N Prion Protein. PLoS Pathog..

[73] Cassard H., Huor A., Espinosa J.C., Douet J.Y., Lugan S., Aron N., Vilette D., Delisle M.B., Marín-Moreno A., Peran P. (2020). Prions from Sporadic Creutzfeldt-Jakob Disease Patients Propagate as Strain Mixtures. mBio.

[74] Kobayashi A., Iwasaki Y., Takao M., Saito Y., Iwaki T., Qi Z., Torimoto R., Shimazaki T., Munesue Y., Isoda N. (2019). A Novel Combination of Prion Strain Co-Occurrence in Patients with Sporadic Creutzfeldt-Jakob Disease. Am. J. Pathol..

[75] Saunders S.E., Bartz J.C., Bartelt-Hunt S.L. (2009). Influence of Prion Strain on Prion Protein Adsorption to Soil in a Competitive Matrix. Environ. Sci. Technol..

[76] Schutt C.R., Bartz J.C. (2008). Prion Interference with Multiple Prion Isolates. Prion.

[77] Sigurdson C.J., Bartz J.C., Glatzel M. (2019). Cellular and Molecular Mchanisms of pion dsease. Annu. Rev. Pathol..

[78] Sigurdson C.J., Nilsson K.P., Hornemann S., Manco G., Polymenidou M., Schwarz P., Leclerc M., Hammarström P., Wüthrich K., Aguzzi A. (2007). Prion Strain Discrimination Using Luminescent Conjugated Polymers. Nat. Methods.

[79] Saunders S.E., Bartelt-Hunt S.L., Bartz J.C. (2012). Occurrence, Transmission, and Zoonotic Potential of Chronic Wasting Disease. Emerg. Infect. Dis..

[80] Barria M.A., Balachandran A., Morita M., Kitamoto T., Barron R., Manson J., Knight R., Ironside J.W., Head M.W. (2014). Molecular Barriers to Zoonotic Transmission of Prions. Emerg. Infect. Dis..

[81] CIDRAP. Center for Infectious Disease Research and Policy. https://www.cidrap.umn.edu/.

[82] Dormann C., Gruber B., Fruend J. (2008). Introducing the Bipartite Package: Analysing Ecological Networks. R News.

[83] Csárdi G., Nepusz T. (2006). The Igraph Software Package for Complex Network Research. Comput. Sci. Eng..

[84] Robinson S.J., Samuel M.D., O’Rourke K.I., Johnson C.J. (2012). The Role of Genetics in Chronic Wasting Disease of North American Cervids. Prion.

[85] Le Dur A., Lai T.L., Stinnakre M.G., Laisne A., Chenais N., Rakotobe S., Passet B., Reine F., Soulier S., Herzog L. (2017). Divergent Prion Strain Evolution Driven by PrP(C) Expression Level in Transgenic Mice. Nat. Commun..

[86] Meade-White K., Race B., Trifilo M., Bossers A., Favara C., Lacasse R., Miller M., Williams E., Oldstone M., Race R. (2007). Resistance to Chronic Wasting Disease in Transgenic Mice Expressing a Naturally Occurring Allelic Variant of Deer Prion Protein. J. Virol..

[87] Silva C.J., Erickson-Beltran M.L., Duque Velásquez C., Aiken J.M., McKenzie D. (2020). A General Mass Spectrometry-Based Method of Quantitating Prion Polymorphisms from Heterozygous Chronic Wasting Disease-Infected Cervids. Anal. Chem..

[88] Hannaoui S., Schatzl H.M., Gilch S. (2017). Chronic Wasting Disease: Emerging Prions and Their Potential Risk. PLoS Pathog..

[89] O’Rourke K.I., Spraker T.R., Zhuang D., Greenlee J.J., Gidlewski T.E., Hamir A.N. (2007). Elk with a Long Incubation Prion Disease Phenotype Have a Unique PrPd Profile. Neuroreport.

[90] Moore S.J., Vrentas C.E., Hwang S., West Greenlee M.H., Nicholson E.M., Greenlee J.J. (2018). Pathologic and Biochemical Characterization of PrP. BMC Vet. Res..

[91] Kong Q., Huang S., Zou W., Vanegas D., Wang M., Wu D., Yuan J., Zheng M., Bai H., Deng H. (2005). Chronic Wasting Disease of Elk: Transmissibility to Humans Examined by Transgenic Mouse Models. J. Neurosci..

[92] Moore J., Tatum T., Hwang S., Vrentas C., West Greenlee M.H., Kong Q., Nicholson E., Greenlee J. (2020). Novel Strain of the Chronic Wasting Disease Agent Isolated from Experimentally Inoculated Elk with LL132 Prion Protein. Sci. Rep..

[93] Wolfe L.L., Fox K.A., Miller M.W. (2014). “Atypical” Chronic Wasting Disease in PRNP Genotype 225FF Mule Deer. J. Wildl. Dis..

[94] Angers R., Christiansen J., Nalls A.V., Kang H.E., Hunter N., Hoover E., Mathiason C.K., Sheetz M., Telling G.C. (2014). Structural Effects of PrP Polymorphisms on Intra- and Interspecies Prion Transmission. Proc. Natl. Acad. Sci. USA.

[95] Baeten L.A., Powers B.E., Jewell J.E., Spraker T.R., Miller M.W. (2007). A Natural Case of Chronic Wasting Disease in a Free-Ranging Moose (Alces Alces Shirasi). J. Wildl. Dis..

[96] Haley N.J., Hoover E.A. (2015). Chronic Wasting Disease of Cervids: Current Knowledge and Future Perspectives. Annu. Rev. Anim. Biosci..

[97] Soldevila M., Calafell F., Andrés A.M., Yagüe J., Helgason A., Stefánsson K., Bertranpetit J. (2003). Prion Susceptibility and Protective Alleles Exhibit Marked Geographic Differences. Hum. Mutat..

[98] Zlotnik I., Rennie J.C. (1963). Further Observations on the Experimental Transmission of Scrapie from Sheep and Goats to Laboratory Mice. J. Comp. Pathol..

[99] Zlotnik I., Rennie J.C. (1965). Experimental Transmission of Mouse Passaged Scrapie to Goats, Sheep, Rats and Hamsters. J. Comp. Pathol..

[100] Kimberlin R. (1976). Slow Virus Diseases of Animals and Man. North-Holland Research Monographs.

[101] Ernst S., Nonno R., Langeveld J., Andreoletti O., Acin C., Papasavva-Stylianou P., Sklaviadis T., Acutis P.L., van Keulen L., Spiropoulos J. (2024). Characterisation of European Field Goat Prion Isolates in Ovine PrP Overexpressing Transgenic Mice (Tgshp IX) Reveals Distinct Prion Strains. Pathogens.

[102] Nonno R., Marin-Moreno A., Carlos Espinosa J., Fast C., Van Keulen L., Spiropoulos J., Lantier I., Andreoletti O., Pirisinu L., Di Bari M.A. (2020). Characterization of Goat Prions Demonstrates Geographical Variation of Scrapie Strains in Europe and Reveals the Composite Nature of Prion Strains. Sci. Rep..

[103] Marin-Moreno A., Aguilar-Calvo P., Espinosa J.C., Zamora-Ceballos M., Pitarch J.L., Gonzalez L., Fernandez-Borges N., Orge L., Andreoletti O., Nonno R. (2021). Classical Scrapie in Small Ruminants Is Caused by at Least Four Different Prion Strains. Vet. Res..

[104] NC1209. NC1209: North American Interdisciplinary Chronic Wasting Disease Research Consortium. https://www.cwd-research.com/home.

[105] Barria M.A., Libori A., Mitchell G., Head M.W. (2018). Susceptibility of Human Prion Protein to Conversion by Chronic Wasting Disease Prions. Emerg. Infect. Dis..

[106] Benavente R., Reed J.H., Lockwood M., Morales R. (2023). PMCA Screening of Retropharyngeal Lymph Nodes in White-Tailed Deer and Comparisons with ELISA and IHC. Sci. Rep..

[107] Alam P., Hoyt F., Artikis E., Soukup J., Hughson A.G., Schwartz C.L., Barbian K., Miller M.W., Race B., Caughey B. (2024). Cryo-EM Structure of a Natural Prion: Chronic Wasting Disease Fibrils from Deer. Acta Neuropathol..

